# The *Drosophila *immunoglobulin gene *turtle *encodes guidance molecules involved in axon pathfinding

**DOI:** 10.1186/1749-8104-4-31

**Published:** 2009-08-17

**Authors:** Bader Al-Anzi, Robert J Wyman

**Affiliations:** 1 Division of Biology 156-29, California Institute of Technology, Pasadena, CA 91125, USA; 2 MCDB, Yale University, KBT 610, 266 Whitney Avenue New Haven, CT 06511, USA

## Abstract

**Background:**

Neuronal growth cones follow specific pathways over long distances in order to reach their appropriate targets. Research over the past 15 years has yielded a large body of information concerning the molecules that regulate this process. Some of these molecules, such as the evolutionarily conserved netrin and slit proteins, are expressed in the embryonic midline, an area of extreme importance for early axon pathfinding decisions. A general model has emerged in which netrin attracts commissural axons towards the midline while slit forces them out. However, a large number of commissural axons successfully cross the midline even in the complete absence of netrin signaling, indicating the presence of a yet unidentified midline attractant.

**Results:**

The evolutionarily conserved Ig proteins encoded by the *turtle*/*Dasm1 *genes are found in *Drosophila*, *Caenorhabditis elegans*, and mammals. In *Drosophila *the *turtle *gene encodes five proteins, two of which are diffusible, that are expressed in many areas, including the vicinity of the midline. Using both molecular null alleles and transgenic expression of the different isoforms, we show that the *turtle *encoded proteins function as non-cell autonomous axonal attractants that promote midline crossing via a netrin-independent mechanism. *turtle *mutants also have either stalled or missing axon projections, while overexpression of the different *turtle *isoforms produces invasive neurons and branching axons that do not respect the histological divisions of the nervous system.

**Conclusion:**

Our findings indicate that the turtle proteins function as axon guidance cues that promote midline attraction, axon branching, and axonal invasiveness. The latter two capabilities are required by migrating axons to explore densely packed targets.

## Background

Genetic studies in *Caenorhabditis elegans *and *Drosophila *have been an effective means of identifying evolutionarily conserved molecular regulators of axonal growth cone guidance. So far, these studies have identified components of the four major guidance cue systems (the netrins, slits, semaphorins, and ephrins) and a variety of morphogens [1-13].

One of the areas where axons make a major decision on their projection path is the embryonic midline [1-13]. Embryonic midline cells are the main source of secreted midline guidance cues encoded by the *netrin *and *slit *genes [2,5,7,9-13]. Netrin is thought to be responsible for attracting commissural axons toward the midline, while slit is responsible for repulsing them. In *Drosophila*, commissural axons initially express high levels of a gene called *commissureless *(*comm*), which functions as an inhibitor of the slit receptor roundabout (robo), thus making those axons sensitive only to netrin and not slit signaling. As soon as the axons reach the midline, *comm *gene expression levels are quickly down-regulated, making those axons responsive to midline slit, which forces them to exit the midline contralaterally [2,11,12]. However, the observation that commissural axons still orient normally and that large numbers of them can reach and cross the midline successfully even in the complete absence of netrin signaling indicates the presence of a yet unidentified midline attractant system [9,10,13].

The conserved turtle/Dasm1 Ig proteins are found in *Drosophila*, *C. elegans*, and mammals [14-18]. Mammalian Dasm1 has been implicated in the dendritic arborization and synaptic maturation of hippocampal neurons [17,18], while the *Drosophila turtle *gene (*tutl*)is thought to be involved, by yet unspecified mechanisms, in coordinating larval motor function [14]. The role of the *C. elegans turtle *homologue, *SSD1.1*/*Igcm-2*, is not yet known [16].

We reexamined the function of the *Drosophila turtle *gene using transgenic overexpression and analysis of both previously isolated and newly generated mutant alleles. Our analysis indicates that *turtle *encodes extracellular molecules that function as midline attractants, and are independent of netrin or slit signaling. We also report that the *turtle *encoded proteins are potent stimulators of axonal branching and invasiveness, thus adding them to the brief list of molecules that function in regulating axonal morphology and guidance.

## Results

### Generating a molecular null of the *turtle *gene

**Figure 1:**
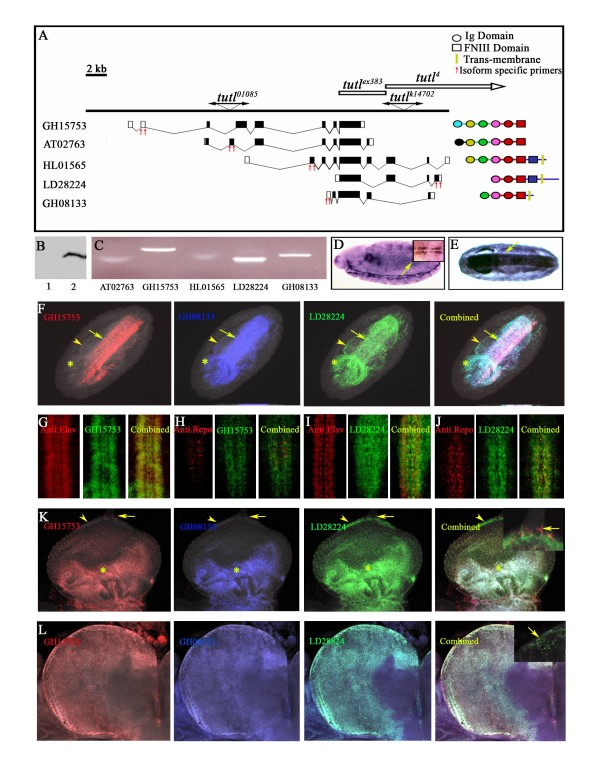
**Genomic organization and *in situ *expression of the *turtle *gene**. **(A) **Schematic diagram showing intron-exon structure of *turtle *transcripts, the proteins that they encode (color-coded to show only clearly defined protein domains shared between the different isoforms), the span of the *tutl*^*ex383 *^and *tutl*^*4 *^deletion, and the two P-element insertions. **(B) **Western blot against the His-tagged diffusible GH015753-encoded isoform of turtle protein expressed in S2 cells. After transfer, both samples were examined on the filter by Ponceau S staining, and although there was abundant protein in both lanes, most of the His-tagged protein was found in the supernatant (2), not the cell pellet (1). Similar results were obtained using the His-tagged AT02763-encoded diffusible isoform of turtle protein (data not shown). **(C) **RT-PCR showing low expression of the AT0276 and HL01565 isoforms in stage 12/13 embryos compared to GH015753, LD2884, and GH08133 isoforms. **(D, E) **RNA *in situ *hybridization against the domains shared by all isoforms indicates that *turtle *is initially expressed close to the midline in embryonic stages 12 to 13 (D, arrow), with later expression spreading throughout the central nervous system (E, arrow). **(F) **RNA *in situ *hybridization of GH015753 (red), LD2884 (green), and GH08133 (blue) isoforms indicates overlapping nervous system expression in stage 13/14 embryos (arrow), with some expression of LD28224 and GH08133 in salivary gland (asterisk) and gut (arrowhead). **(G-J) **Staining with the neuronal marker anti-Elav (G, I, red) indicates that both GH015753 and LD2884 (G-I, green) are expressed in neuronal cells. Both isoforms also co-localized with some anti-Repo-positive glial cells (H, J, red). **(K) ***RNA in situ *hybridization in wandering third larval instar eye discs indicates that all isoforms are expressed in the morphogenic furrow (asterisk), with some cells at the tip of the eye disc expressing only GH015753 (arrow), which are surrounded by cells expressing only LD28224 (arrowhead). **(L) ***RNA in situ *hybridization in wandering third larval instar brains indicates strong, overlapping expression of the *turtle *isoforms throughout the optic lobe, with some cells in the optic lobe-retinal nerve junction expressing only the LD2884 isoform (arrow).

In the initial *turtle *gene publication, the exon-intron map provided by the authors indicates that none of the generated deficiencies removes all *turtle *transcripts [14]. The potentially diffusible isoforms encoded by expressed sequence tag (EST) clones AT02763 and GH15753 near the 5'-end of the gene are still present even in the largest deletion, *tutl*^*4*^. With this in mind, we mobilized the *tutl*^*k14703 *^P-element insertions to generate null mutation of the *turtle *gene (Figure 1A). A newly generated allele, *tutl*^*ex383*^, is embryonic lethal and fails to complement the adult lethality of the previously identified *turtle *mutant allele *tutl*^*01085*^. Southern blotting, PCR, and DNA sequencing indicate that this allele contains an 11,430 base pair (bp) deficiency within the *turtle *gene that should disrupt all transcripts (Additional file 1). Staining with a variety of cell fate markers indicates that the embryonic lethality of *tutl*^*ex383 *^is not due to a defect in cell fate induction (Additional file 2).

### *turtle *is expressed in areas critical for axon pathfinding decisions and encodes a diffusible isoform

A Blast search using *turtle *genomic sequence indicated that the gene encodes five different isoforms. The isoforms encoded by EST clones AT02763 and GH157753 do not contain any hydrophobic membrane-spanning domains, suggesting that they may be diffusible proteins, while the isoforms encoded by the EST clones HL010565, LD28824, and GH08133 have a membrane-spanning domain, indicating that they are membrane bound.

To determine whether the proteins encoded by AT02763 and GH157753 are secreted, we transiently transfected *Drosophila *Schneider 2 (S2) cells with carboxy-terminal His-tagged versions of these transcripts. Western blots against the His tag indicate that most of the tagged proteins were present in the medium fraction and not in the cell pellet, confirming that they are secreted (Figure 1B).

Using isoform-specific primers and RT-PCR, we detected the expression of all the *turtle *isoforms in embryos during the start of neuronal axonogenesis (embryonic stages 12 to 13; Figure 1C). However, the expression levels of AT02763 and HL010565 were much lower than those of GH157753, LD28824, and GH08133.

We made anti-sense Dig-labeled RNA *in situ *hybridization probes against the exons encoding the fifth immunoglobulin domain and the first Fibronectin III domain of *turtle*. During stages 12 and 13, when neurons begin to project axons, expression was initially restricted to a segmentally repeated cluster of cells surrounding the midline (Figure 1D). Later, expression spread throughout the central nervous system (Figure 1E).

We also generated RNA probes against sequences that are unique to each isoform. Each probe type was labeled with a different fluorescent tag. RNA *in situ *hybridization with GH15753, LD28824, and GH08133 isoforms in wild-type embryos indicated strong, overlapping nervous system expression during stages 13 to 14 (Figure 1F, arrow). We also observed overlapping expression of LD28824 and GH08133 in certain areas of the gut and salivary glands (Figure 1F, arrowhead and asterisks, respectively). However, RNA *in situ *hybridization performed against the AT02763 and HL010565 isoforms did not produce a signal above background (data not shown), thus further confirming their low expression levels.

We were unable to detect fluorescence signal of any isoform in early stage 12 embryos. A possible explanation is that fluorescence probes are too short to give a detectable signal in stages when *turtle *levels are low; indeed, like other cell recognition molecules, *turtle *transcription might be up-regulated in later embryonic stages, further enhancing *in situ *detection.

To further clarify the expression pattern of the *turtle *isoforms within the nervous system, we performed RNA *in situ *hybridization with GH15753- or LD28824-specific probes on embryos stained with the neuronal marker anti-Elav or non-midline glia marker anti-Repo. Both isoforms are largely expressed in segmentally repeated neurons both near to and distant from the midline, with some minor expression in glia (Figure 1G-J).

In wandering third instar larvae, RNA *in situ *hybridization of imaginal discs and brains showed robust and partially overlapping expression of GH15753, LD28824, and GH08133. In the eye disc, GH15753, LD28824, and GH08133 are expressed within the morphogenetic furrow (Figure 1K, asterisks). However, in the tip of the eye disc, where the optic stalk is located, we observed a cluster of cells that are only GH15753-positive (Figure 1K, arrow). Those cells were surrounded by a group of cells that express only LD28824 (Figure 1K, arrowhead). In the brain, strong and overlapping expression of GH15753, LD28824, and GH08133 was observed in the optic lobe (Figure 1L).

However, in the region where the optic stalk contacts the optic lobe, we noticed a cluster of cells that express only LD28824 (Figure 1L inset, arrow).

### *turtle *is involved in midline crossing

**Figure 2:**
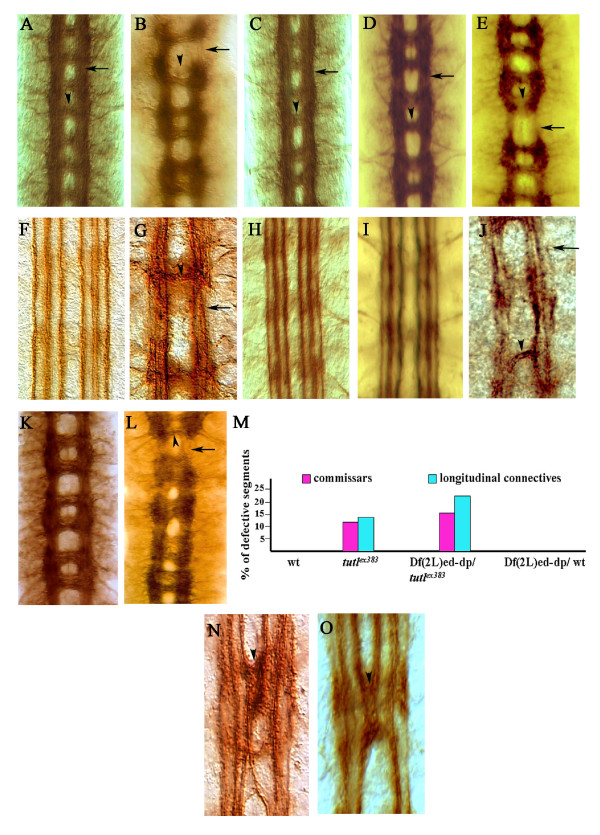
***turtle *is involved in promoting midline crossing**. **(A) **Wild-type embryonic longitudinal connectives (arrow) and commissures (arrowhead) stained with BP102 antibody. **(B) ***tutl*^*ex383 *^homozygous embryos have fragmented connectives and missing commissures (arrowhead and arrow, respectively). **(C, D) **Both Elav-Gal4 (C) and Sim-Gal4 (D) expression of the different *turtle *isoforms fully rescues the *tutl*^*ex383 *^longitudinal connective (arrow) and commissure (arrowhead) defects. **(E) **No rescue of those phenotypes is observed with non-midline glia driver Repo-Gal4. **(F) **The ventral nerve cord in wild-type embryos has FasII-positive fascicles that are well-formed and do not cross the midline. **(G) ***tutl*^*ex383 *^homozygous embryos have FasII-positive fascicles that are fragmented (arrowhead), with axons that cross the midline (arrow). **(H, I) **Elav-Gal4 (H) and Sim-Gal4 (I) expression of the different *turtle *isoforms fully rescues the *tutl*^*ex383 *^FasII-positive fascicle defects, preventing fascicles from fragmenting or crossing the midline. **(J) **No rescue of longitudinal connectives (arrow) and commissures (arrowhead) phenotypes is observed with non-midline glia driver Repo-Gal4. **(K-M) **Df(2L)ed-dp/wild type (wt) embryos have normal commissures and longitudinal connective (K, M); however, embryos trans-heterozygous for the same genomic deficiency over the *tutl*^*ex383 *^allele in Df(2L)ed-dp/*tutl*^*ex383 *^have commissure and longitudinal (arrowhead and arrow, respectively) connective defects that are similar to those of *tutl*^*ex383 *^homozygous embryos (L, M). **(N) **Sca-Gal4 pan-neuronal overexpression of only the diffusible *turtle *isoforms causes entire innermost FasII-positive fascicles to cross the midline (arrowhead. **(O) **Sim-Gal4 overexpression of the diffusible *turtle *isoforms produces similar defects (arrowhead) to Sca-Gal4, even though, in this case, *turtle *is expressed only in the midline cells and not in FasII-positive tracks.

In the late embryonic stages, most central nervous system axons are arranged in two longitudinal connectives that are linked in each body segment by two midline-crossing commissures. These structures can be easily visualized using the BP102 antibody (Figure 2A). We noticed that close to 10% of *tutl*^*ex383 *^embryonic segments have gaps in the longitudinal connectives and commissures (Figure 2B, M), a defect that is typically observed in embryos that lack *netrin *or the genes for the Netrin receptor Frazzled [9,10,19]. A minor enhancement of this phenotype was observed in embryos that are tarns-heterozygous *tutl*^*ex383 *^over a genomic deficiency Df(2L)ed-dp that removes the *tutl *gene, thus indicating that this mutant allele is almost a genomic null (Figure 2L, M).

The anti-Fasciclin (Fas)II antibody stains three longitudinal axon fascicles on each side of the midline (Figure 2F). In 60% of homozygous *tutl*^*ex383 *^embryos, these axons are defasciculated, and some inappropriately project to the midline (Figure 2G). This phenotype is distinct from that of mutations that cause a reduction in midline repulsion, such as *robo *or *slit*. In *robo *mutants, the innermost fascicles remain intact but repeatedly crisscross the midline [2,11,12], while in *tutl*^*ex383 *^embryos the FasII tracks maintain their relative distance from the midline, but axons emanating from all three fascicles bundle together as they cross the midline (Figure 2G).

To confirm that the observed defects are due to loss of *turtle *function, we performed complementation tests and transgenic rescue. Embryos trans-heterozygous for *tutl*^*ex383 *^and *tutl*^*01085 *^have defects in commissures, longitudinal connectives, and FasII-tracks that are similar to those of *tutl*^*ex383 *^homozygotes. However, these defects occur with a lower frequency and severity than is observed in *tutl*^*ex383 *^embryos (data not shown). Post-mitotic neuronal expression of any of the different *turtle *isoforms in a *tutl*^*ex383 *^background, using either Elav-Gal4 or the midline cell driver singleminded-Gal4 (Sim-Gal4), fully rescues these midline crossing defects (Figure 2C, D, H, I). Furthermore, transgenic expression using the pan-neuronal Elav-Gal4 driver of the diffusible isoform encoded by the EST clone AT02763 on a *tutl*^*ex383 *^mutant background is sufficient to rescue adult lethality even though this isoform is weakly expressed in the wild type.

Both Elav-Gal4 and Sim-Gal4 are expressed close to or within the midline. If *turtle *expression near the midline is critical for its axonal midline crossing functions, then its expression in cells that are away from the midline, using Repo-Gal4, should not rescue the mutant phenotype. Indeed, mutant *tutl*^*ex383 *^embryos that transgenically express *turtle *in only Repo-Gal4-positive glia do not show any rescue (Figure 2E, J). These results indicate that mutation in the *turtle *gene is indeed responsible for the midline crossing defects and lethality seen in *tutl*^*ex383 *^embryos, and that *turtle *expression close to or within the midline is most important for this function.

We also performed pan-neuronal overexpression of the different isoforms in a wild-type background using a Sca-Gal4 driver. While no obvious phenotype resulted from overexpression of the membrane-bound isoforms, overexpression of the diffusible isoforms produces FasII-tracks that crisscross the midline in a manner reminiscent of *robo *mutants (Figure 2N), a phenotype that is typically interpreted as an increase in midline attraction [2,11,12,20-25]. The same phenotype was also produced when the diffusible isoforms were expressed only in the midline cells and not in the FasII-positive axons using Sim-Gal4 (Figure 2O), which indicates that the secreted *turtle *isoforms can produce their effects on midline axons via a non-cell autonomous mechanism.

The above results, combined with *turtle *expression near the midline during critical stages of axon development, implicate *turtle *in increasing axonal midline crossing. All isoforms are fully capable of rescuing the axon midline crossing defects in *turtle *mutants. However, the diffusible isoforms are the most potent as they are the only isoforms that induce a gain-of-function phenotype when overexpressed on a wild-type background.

### *turtle *promotes midline crossing via a *netrin*- and *slit*-independent mechanism

Turtle proteins may promote midline attraction by one of three potential mechanisms. They may function as part of the netrin pathway, mediating netrin signaling. Alternatively, they may directly attract axons via their own unique pathway. Finally, they may function as inhibitors of the midline repellent slit pathway, which would then produce an indirect increase in the midline attraction signal.

**Figure 3:**
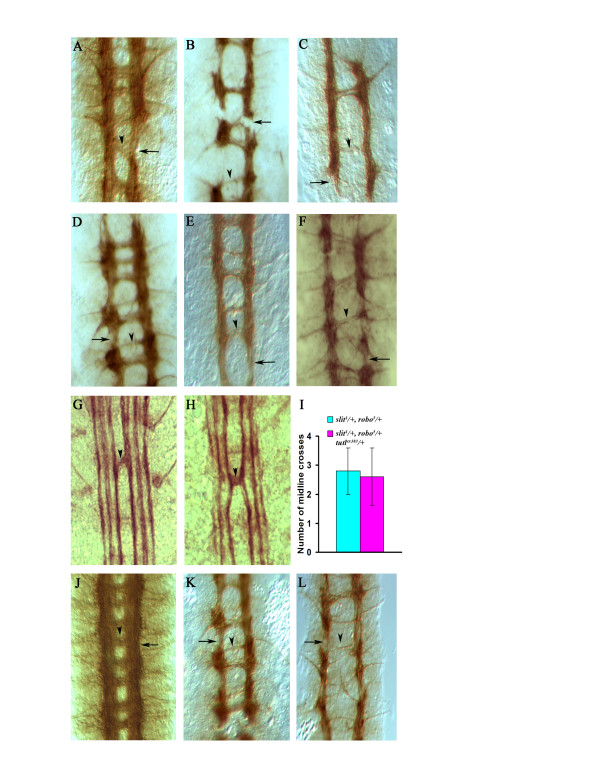
**The *turtle *gene products act as *netrin*-independent midline attractants**. **(A) ***Df*(*1*)*NP-5 *homozygous embryos are missing the *netA *and *netB *genes and, consequently, exhibit gaps in their longitudinal connectives (arrow) and fragmented commissures (arrowhead), as revealed by BP102 staining. **(B, C) ***Df*(*1*)*NP-5; tutl*^*ex383*^/+ embryos show an enhancement of both defects (B, arrow and arrowhead), while *Df*(*1*)*NP-5; tutl*^*ex383 *^double mutants have an extreme reduction in commissures, with only one thin fragmented commissure formed throughout the length of the embryo (C). **(D) ***fra*^*GA957 *^homozygous embryos exhibit gaps in their longitudinal connectives (arrow) and fragmented commissures (arrowhead) as revealed by BP102 staining. **(E)***fra*^*GA957*^, *tutl*^*ex383*^/+ embryos show an enhancement of both defects. while *fra*^*GA957*^,*tutl*^*ex383 *^double mutants have an extreme reduction in commissures (arrowhead) **(G) ***slit*^*1*^/+, *robo*^*5*^/+ embryos have a reduction in *slit *signaling that can produce a minor midline crossing defect revealed by FasII staining (arrowhead). **(H, I) ***slit*^*1*^/+, *robo^*5*^*/+, *tutl*^*ex383*^/+ triple heterozygotes do not show any enhancement nor suppression of the FasII axon crossing defect compared to *slit*^*1*^/+, *robo*^*5*^/+ double heterozygotes alone. **(J) ***abl*^*1 *^homozygous embryos do not show any defect in commissure or longitudinal connectives as revealed by BP102 staining (arrow and arrowhead). **(K, L) ***tutl*^*ex383*^; *abl*^*1*^/+ embryos show an enhancement of commissural defects (K), while *tutl*^*ex383*^; *abl*^*1 *^double mutants have an extreme reduction in commissure formation (L, arrow) and fragmented longitudinal connectives (L, arrowhead).

Null mutations in essential genes in the same pathway do not modify each other's phenotype in double homozygous configurations, while genes that affect the same biological process but are part of different and redundant pathways do in both heterozygous and double homozygous combinations. If turtle directly facilitates netrin function, then reducing turtle levels in embryos with no netrin signaling should produce no additional modifications of those embryos' mutant phenotype, since there is no netrin signaling in the first place to be modified. However, if turtle functions in a parallel pathway that induces midline attraction via a netrin-independent mechanism, then reduction in turtle signaling should produce a synergistic effect that further enhances *netrin *or *frazzled *mutant phenotypes. The fly genome contains two *netrin *genes, *netA *and *netB*, and one *frazzled *gene (*fra*). The genomic deficiency *Df*(*1*)*NP-5*, which removes both *netrin *genes and the *fra *mutant allele *fra*^*GA957*^, results in embryos that have fragmented commissures and gaps in longitudinal connectives thought to reflect a complete lack of netrin pathway signaling [9,10] (Figure 3A, D). The removal of even one copy of the *turtle *gene dramatically enhances the *netrin *or *fra*^*GA957*^mutant phenotype (Figure 3B and 3E, respectively), while the complete removal of both turtle and netrin signaling in *Df*(*1*)*NP-5; tutl*^*ex383 *^or *fra*^*GA957*^, *tutl*^*ex383 *^double homozygotes produces embryos that typically have only one thin and fragmented commissure along the entire length of the embryo (Figure 3C and 3F, respectively), a phenotype that bears similarities to that of the *robo *down-regulator *comm *[1,12]. This synergistic enhancement of *turtle *mutation in embryos that lack netrin signaling indicates that turtle promotes midline attraction via a netrin-independent mechanism, and not as part of the netrin signaling pathway.

In *Drosophila*, the removal of the *slit *gene produces embryos with commissural axons that fail to exit the midline, while the loss of the slit receptor *robo *produces a less severe midline phenotype. This is due to the presence of two other *robo*-like *slit *receptors that can partially compensate for *robo *loss-of-function [1,11,20,21]. Double heterozygous *slit*/*+*, *robo*/*+ *embryos have a sufficient drop in slit signaling to produce minor defects in midline crossing revealed by FasII staining [1,11,20,21] (Figure 3G). This phenotype can be modified when other components of the slit pathway are genetically removed [11,12,20-25]. If the turtle proteins promote midline crossing by functioning as a slitpathway inhibitor, then the reduction in turtle function should suppress the midline crossing defect seen in *slit*^*1*^/*+*, *robo*^*5*^/*+ *embryos. We did not detect any modification of this defect in *slit*^*1*^/*+*, *robo*^*5*^/*+*, *tutl*^*ex383*^/*+ *embryos (Figure 3H, I), which makes it unlikely that turtle proteins functions as an inhibitor of the slit pathway.

The Abelson tyrosine kinase (abl) is a critical point of convergence for both attractive and repulsive midline signals received by growing axons. The integrative capacity of abl is due to its ability to physically link midline guidance cue receptors, such as the netrin receptor fra and the slit receptor robo, to actin cytoskeleton modulators such as ena and trio [21-24]. The general effect of increased abl signaling on both netrin and slit pathways is to produce an increase in the ability of axons to cross the midline. Genetic null mutations in the *abl *gene alone, such as *abl*^*1*^, produce only minor midline crossing defects. This might be due to the fact the abl protein is maternal loaded by *abl*^*1 *^heterozygous mothers into their homozygous mutant progeny. However, the minor midline crossing defects observed in those embryos can be genetically modified if other components of the *abl *pathway are also reduced. We find that removing one copy of *abl *dominantly enhances the commissural defect seen in *tutl*^*ex383 *^mutant embryos (Figure 3K). Furthermore, *tutl*^*ex383*^; *abl*^*1 *^double homozygous embryos have a commissural axon defect that is similar to that of *netA*,*netB*,*tutl*^*ex383 *^and *fra*^*GA957*^, *tutl*^*ex383*^homozygous embryos (Figure 3L).

All of the above results indicate that the turtle proteins function as a midline attractant via a netrin-independent mechanism that does not involve the suppression of the slit pathway. Due to maternal loading of abl proteins, it is difficult to conclusively determine if turtle signaling functions in an abl-independent or -dependent manner. Nevertheless, these results do indicate that turtle pathway signaling is integrated with other guidance cue pathways to ultimately produce appropriate midline crossing behavior in growing axons.

### *turtle *promotes both motor and retinal axon branching and invasive behavior

**Figure 4:**
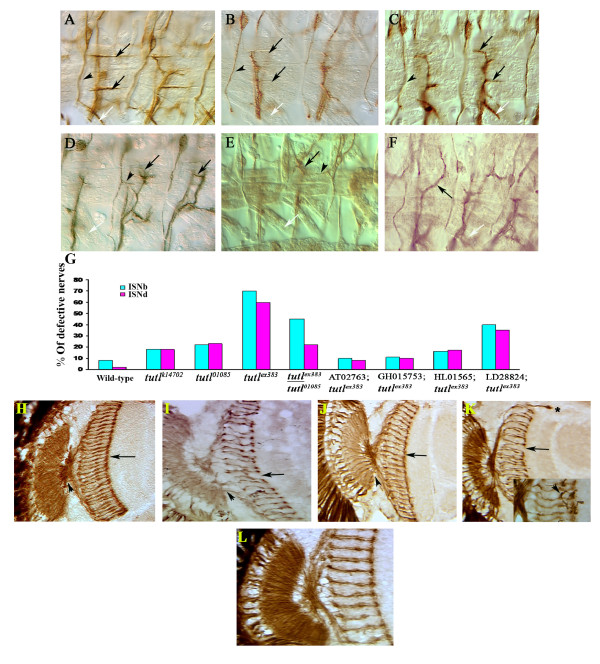
**The *turtle *gene functions non-cell autonomously in promoting motor axon branching**. **(A) **Wild-type embryo stained with FasII antibody, showing the ISNb nerve branch innervating the cleft between muscles 6 and 7 (lower black arrow), muscle 13, and the cleft between muscles 12 and 13 (upper black arrow), the ISNd nerve branch (white arrow), and the TN nerve (arrowhead). **(B) ***tutl*^*ex383 *^mutants have ISNb nerves that frequently fail to project final branches (black arrows), and many segments lack an ISNd (white arrow) but still posses a normal TN nerve (arrowhead). **(C) **Elav-Gal4 expression of the diffusible *turtle *isoform fully rescues both ISNb and ISNd nerve branch defects in *tutl*^*ex383 *^homozygous embryos (arrows and arrowhead). **(D, E) **Both pan-neuronal Sca-Gal4 (D) and pan-skeletal muscle 24B-Gal4 and G14-Gal4(E and D, respectively) overexpression of *turtle *isoforms cause ISNb nerves to either excessively branch (black arrows) Yesor stall, cause the TNs to send out ectopic branches (arrowheads), and produce missing ISNd nerves (white arrows). **(G) **Quantification of the motor nerve defects seen in 55 to 60 A2 to A7 embryonic hemisegments in *turtle *mutants (*tutl*^*k14703*^, *tutl*^*10805*^, and *tutl*^*ex383*^), in complementation testing (*tutl*^*ex383*^/*tutl*^*10805*^), and in *tutl*^*ex383 *^homozygotes with different *turtle *isoforms transgenically expressed using Elav-Gal4. Note that only the two diffusible isoforms rescue the mutant to near-wild type levels of motor axon pathfinding errors. The *turtle *gene functions non-cell autonomously in promoting retinal axon invasiveness and branching. **(H) **Adult wild-type head horizontal section showing retinal axons visualized with 24B10 antibody. Note normal optic chiasm (arrowhead) and regular array of R7 axon terminations (arrow). **(I) ***tutl*^*k14703 *^adult mutants have gaps in the R7 termination line (arrow) and an irregular chiasm (arrowhead). **(J) **The retinal axon defects in *tutl*^*ex383*^/*tutl*^*k14703 *^are rescued when the diffusible *turtle *isoforms are transgenically expressed using the pan-neuronal driver Elav-Gal4. **(K) **Eye-specific GMR-Gal4 overexpression of the diffusible *turtle *isoforms in a wild-type background produces retinal axons that invade the cortex (asterisk), gaps in the R7 termination line (arrow), and some axons with extra branches (arrowhead). **(L) ***tutl*^*k14703 *^EGUF/hid mutant eyes have normal optic and R7 projections (L).

In stage 16 wild-type embryos, the ventral body wall muscles are innervated by an invariant pattern of motor axons coming from ISNb, SNc, and ISNd nerves [3,26] (Figure 4A). In *tutl*^*ex383 *^embryos, the ISNb motor axons successfully reach the vicinity of their respective targets. However, the majority of them fail to send one or more of the final axon branches to contact their muscle targets. Half of the hemisegments also lack ISNd nerves (Figure 4B, G). Both *tutl*^*01085 *^and *tutl*^*k14703 *^mutant embryos exhibit a similar phenotype, albeit with a lower frequency (Figure 4G). Since the initial publication on *turtle *reported that there were no motor axon pathfinding defects, we conducted a blind test by an independent observer to confirm our results in *tutl*^*01085*^, one of the initially reported alleles [14]. The observer was able to correctly identify the mutant embryos. Furthermore, his count of the percentage of defects in ISNb and ISNd nerves was similar to our estimate. We therefore conclude that *tutl*^*01085 *^mutants have a robust motor axon pathfinding defect.

Using complementation testing and transgenic rescue, we verified that, like the midline crossing defects, these motor axon pathfinding defects were caused by mutations in *turtle*. Embryos trans-heterozygous for *tutl*^*ex383 *^and *tutl*^*01085 *^have similar embryonic motor axon projection defects to *tutl*^*ex383*^homozygotes (Figure 4G), but with lower frequency. Furthermore, expression of either of the two diffusible *turtle *isoforms via a Elav-Gal4 driver in *tutl*^*ex383 *^homozygotes can rescue these defects (Figure 4C, G). However, the motor axon projection defect is only reduced, rather than fully rescued, by the two membrane-spanning isoforms (Figure 3G), which further points to the strong potency of the diffusible isoforms in mediating *turtle*'s function.

We examined the consequences of neuronal overexpression of the different *turtle *isoforms in a wild-type background using the pan-neuronal driver Sca-Gal4. This overexpression produced excessive branching and stalling of motor nerves, with some nerves, such as the transverse nerve (TN), innervating muscles that are not their normal targets (Figure 4D). The diffusible isoforms were, once again, more potent in producing axonal abnormalities when overexpressed than the membrane-bound isoforms (Additional file 3).

Even though *turtle *expression is largely restricted to the nervous system during development, some isoforms are secreted, suggesting that they may function as extracellular signaling molecules rather than as cell autonomous receptors. Therefore, one would predict that ectopic *turtle *overexpression solely in axonal targets, such as body wall muscle, should produce an alteration in motor axon morphology similar to that observed when *turtle *is overexpressed in the motor neurons themselves. When the different turtle transgens were expressed in body wall muscles using 24B-Gal4 and G14-Gal4 drivers, we indeed observed motor axon defects similar to those seen using the neuronal driver Sca-Gal4 (Figure 4E, F), even though the axons in this case expressed wild-type levels of *turtle*.

In wild-type *Drosophila *adult eyes the retinal axons are arranged in a regular array, with photoreceptor R7 axon terminations forming a solid line across the M6 layer of the medulla [27] (Figure 4H). In the weak mutant allele *tutl*^*k14703*^, approximately 20% of the homozygotes reach the adult stage; these adults have an optic chiasm that is disorganized and gaps in the R7 termination line (Figure 4I). Expression of the diffusible isoform using the pan-neuronal driver Elav-Gal4 in *tutl*^*ex383*^/*tutl*^*k14703 *^rescues most aspects of this defect (Figure 4J). Overexpression of the different *turtle *isoforms in a wild-type background using the retina-specific GMR-Gal4 driver produces abnormalities that are strikingly similar to defects seen in *turtle*-overexpressing motor axons, in that many retinal axons stall before reaching their targets and some sprout extra axonal processes (Figure 4K, arrow and arrowhead, respectively). The diffusible isoforms were, again, more potent in producing these axonal abnormalities when overexpressed than the membrane-bound isoforms.

As noted above, *turtle *is expressed in both the eye disc and the contact zone between the optic lobe and optic stalk in third instar larvae. If *turtle *functions as a signaling molecule that guides retinal axons to their optic lobe targets, the critical domain of expression should be the optic lobe rather than the eye disc. To test this, flies with eyes composed of *turtle *mutant cells and brains expressing wild-type levels of *turtle *were generated using the eyeless-GAL4 UAS-FLP (EGUF)/hid mosaic technique [28]. Although the resulting eyes were smaller than wild-type, they had normal optic chiasma and R7 projections (Figure 4L), indicating that the source of retinal axon defects in *tutl*^*k14703 *^mutants is reduced *turtle *levels in the optic lobe, rather than in the retinal axons. These results indicate that, in retinal axons, *turtle *does not function as part of a guidance receptor complex, nor as a homophilic or hetrophilic cell adhesion molecule that is needed in both retinal axons and their brain targets.

**Figure 5:**
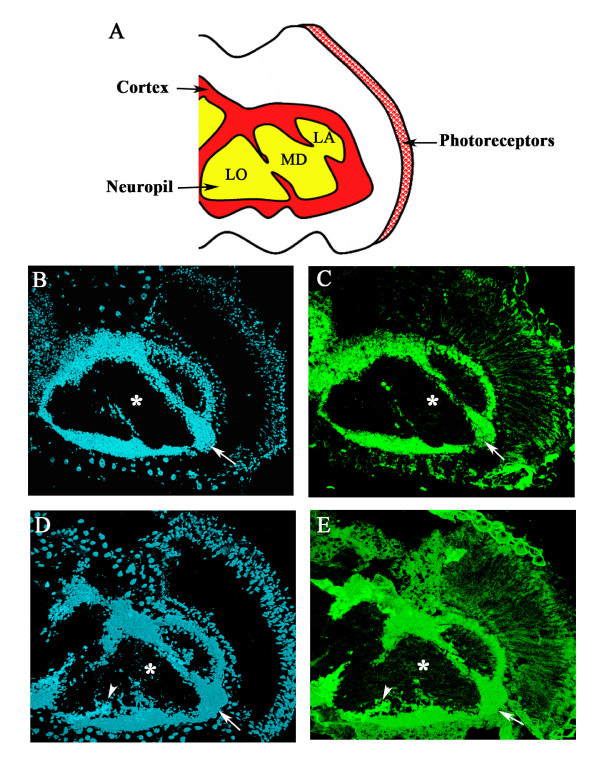
**Pan-neuronal Elav-Gal4 overexpression of the diffusible *turtle *isoform AT02763 promotes neuronal invasiveness**. **(A) **Representation of the major histological divisions of the adult fly nervous system in the head (LA, lamella; LO, lobula; MD medulla). **(B) **Wild-type head horizontal section, stained with the nuclear marker DAPI (blue fluorescence), showing the peripheral location of the cortex (arrow) and the central location of DAPI-free neuropil (asterisk). **(C) **Adult horizontal section stained with the neuronal marker Elav (green fluorescence), showing that most of the cortex is composed of neuronal cells (arrow). **(D) **Pan-neuronal overexpression of the diffusible isoform AT02763 produces cells that invade the normally cell-free neuropil layer (arrowhead)s. **(E) **Most of these invasive cells are Elav-positive neurons (arrowhead).

The fly and vertebrate central nervous systems share a common two-layered organization when viewed under histological staining (Figure 5A). The fly cortex is exclusively composed of neuronal cell bodies, while the deep layer, or neuropil, is largely composed of axons and neurites (Figure 5A). Overexpression of the diffusible *turtle *isoforms in a wild-type background produces neuronal structures that do not conform to these histological boundaries. Diffusible *turtle *isoform overexpressing retinal axons occasionally invade the cortex (Figure 4K, asterisks), and, conversely, *turtle *overexpressing neuronal cell bodies invade the normally cell-free neuropil layer (Figure 5D, E).

Based on the results obtained from both tissue-specific transgenic expression and EGUF/hid mutant eye analysis, we conclude that some *turtle *isoforms can function as extracellular signaling molecules that act via a non-cell autonomous mechanism to promote axonal branching and invasive behavior.

## Discussion

We propose that the *turtle *gene encodes a midline attractant. This conclusion is based on the observation that *turtle *null mutants have a reduction in the number of midline-crossing commissures, while overexpression of diffusible *turtle *isoforms causes axons that normally do not cross the midline to do so. This conclusion is further supported by the initial expression of *turtle *isoforms close to the midline during embryonic stages where axons initiate their midline-crossing behavior.

Genetic interactions indicate that *turtle *attracts commissural axons via a netrin-independent mechanism, and does not stimulate midline attraction by direct inhibition of the slit midline repellent signaling pathway. However, a reduction in the level of abl, a known component of both netrin and slit pathways [22-25], does enhance *turtle *midline defects.

Although most of our data suggest that the turtle proteins promote axonal midline crossing, in *turtle *null mutants some FasII-positive tracks do abnormally cross the midline, suggesting that the turtle signal prevents, rather than encourages, midline crossing in some axons. It is possible, therefore, that turtle produces different effects on different axons. Indeed, many guidance cues, such as netrin, have opposite effects on different axons, depending largely, though not entirely, upon which type of netrin receptor complex the axons express [1,29-31]. However, even mutations in genes that function chiefly to increase midline crossing, such as *abl*, are known to produce FasII-positive tracks that cross the midline abnormally [23]. Furthermore, as was stated in the results section above, the manner in which FasII-positive tracks in *turtle *null mutants cross the midline is clearly distinct from the midline crossing defects seen in mutations or transgenic manipulations that are known to reduce midline repulsion.

Our results also indicate that the *turtle *gene encodes signaling molecules that promote axon and neuronal cell body invasiveness and axonal branching, and that these molecules are capable of performing those functions via a non-cell autonomous mechanism. Axon branching and invasiveness are both necessary behaviors for growing axons to explore and choose between closely packed targets, and the loss of these activities could explain the inability of *turtle *mutant axons to make their final connections. Indeed, *turtle *has been re-isolated in a gain-of-function screen by the Zinn group at Caltech for factors that promote motor axon branching in *Drosophila *[32]. It is also worth noting that human *Dasm1 *is frequently overexpressed in tumors [15]. That *turtle *can cause both axons and cell bodies to enter abnormal locations suggests that *turtle *family members may stimulate the loss of tissue organization common in tumors.

In all tissues in which we examined *turtle *function, we found the diffusible isoforms to be more potent both in rescuing the mutant phenotype and in producing a gain-of-function phenotype when ectopically expressed. This point is further illustrated by the fact that the embryonic lethality in *tutl*^*ex383 *^mutants, which carry a deletion that affects all isoforms, can be rescued to the level of adult viability when only the diffusible isoforms are expressed. In contrast, the membrane isoforms are only capable of reducing the severity of some aspects of the *tutl*^*ex383 *^phenotypes. The importance of these diffusible isoforms is further supported by the high degree of homology in the extracellular domains, but not the cytoplasmic domains, of *Turtle*/*Dasm1 *family members. However, it is possible that this increase in potency is due to protein domains shared between those two isoforms that are unique to them.

The *turtle*/*Dasm1 *mammalian homologue is expressed in a pattern that is suggestive of a potential role in axon pathfinding [15]. However, to date, the role of mammalian and *C. elegans *homologues in axon pathfinding has not been examined, nor has their role in vertebrate tumorigenesis been much explored. We hope that our results will draw attention to these novel signaling molecules and lead to further investigation of the downstream pathways triggered by *turtle *signaling.

## Materials and methods

### Stocks

Two P-element insertions in the *turtle *locus, P {ry^+t7.2 ^= PZ} l(2)01085 and P {w^+mc ^= lacW} l(2)k14703, were obtained from the Berkeley *Drosophila *Genome Project, and their flanking genome sequences were determined by the inverse PCR primer method

### Determining the extent of genomic deficiency in *tutl*^*ex383*^

A *Cla*I restriction map was made of the 30 kbp genomic interval where the *turtle *gene is located. A Southern blot of *Cla*I digests using genomic DNA of wild-type, and *tutl*^*l*(2)*k14703*^, and *tutl*^*lex383 *^mutants was performed, and the blot was sequentially probed with two different radio-labeled probes that span different portions of the *turtle *genomic interval. The first probe, which spanned the 3'-end of the transcript, should hybridize to a 6-kb band in wild-type DNA and two different bands in the *tutl*^*l*(2)*k14703*^/CyO DNA: one will be a 6-kb band corresponding to the wild-type copy of *turtle *in the CyO balancer, and the other will be an 8-kb band corresponding to the *turtle *mutant chromosome (since the P-element contains a *Cla*I site 7 kb away from its 5' end). When using the 3'-end probe, a clear *Cla*I polymorphism in *tutl*^*ex383 *^was detected and a new band of approximately 13 kb appeared. This band was not present in either wild-type or *tutl*^*l*(2)*k14703 *^genomic DNA, thus demonstrating that a deficiency was induced in the *turtle *locus (Figure 1A). The second probe flanked the 5'-end of the *turtle *gene. This probe should produce bands of 1.5, 3, 7, and 8 kb in both wild-type and *tutl*^*l*(2)*k14703*^. This probe did not produce any *Cla*I polymorphism in the *tutl*^*ex383 *^DNA digest (Figure 1A). We concluded from the previous results that the deficiency did indeed occur in the *turtle *gene in *tutl*^*ex383 *^and is restricted to the region between the two probes.

To clarify the extent of the deficiency in *tutl*^*ex383*^, we designed two series of PCR primers that spanned the genomic interval around the P-element insertion. These were called primer series A and B. In the A series, we designed six primers that were 2 kbp apart. The first primer, A1, was located approximately 16 kb away from the 5'-end of the P-element insertion while the last primer, A6, was located approximately 6 kb from that site. Series B had only two primers, both located after the 3'-end of the P-element insertion; primer B1 was located 1 kbp away from the site of P-element insertion, while primer B2 was 4 kbp away from that site. A PCR reaction was done on genomic DNA isolated from *tutl*^*ex383 *^with one primer of the A series and one primer of the B series (Additional file 1B). Only 1 minute of elongation time per cycle was given to the PCR reaction. Hence, amplification occurred only if the left and right primers were brought within 1 kb of each other as a result of the deficiency. Only the combination A4/B2 gave a product, which was approximately 900 bp long (Additional file 1C). Since those two primers are about 14 kbp away from each other in the wild type, their ability to produce a PCR product demonstrated a deficiency had removed 12 kbp of genomic DNA within the *turtle *locus in *tutl*^*ex383*^. We then subcloned the PCR product and sequenced it. The sequencing result indicates the presence of an 11,430 bp deficiency in *tutl*^*ex383 *^(Additional file 1D). Such a deficiency is more than enough to effectively remove all the different isoforms of turtle, thereby producing a true null allele of this gene.

The PCR primer series A and B were designed based on the genomic sequence of the P1 Clone DS00830, and have the following sequences: A1, TAC TTT TGC GGA CAC TCT CTC TGT CC; A2, GCT TTG CTG CCT GAC AAA TAG CAA GG; A3, AGG GAA ARC GAA GCC AAA CCG AAA GG; A4, ATA AGA ATC GGA TGC GAA CCG TCA GC; A5, GCC ATG CGA GTT TTA CTG TTC TAG GC; A6, ATC CAG AGT AAC AGA GTC TAC GAG CC; A7, AAT CTG GCT TCA TAG CGC CTT GCA GC; B1, CCA GTG GAG TAT GGC AAT GAG AAT GG; B2, TTT TTC TGG GTG TGA GTT TGC AGG GG.

### RT-PCR, RNA localization, and protein immunocytochemistry

Total RNA was extracted from 1 mg stage 12/13 wild-type embryos using a Qiagen RNA Extraction Kit (Quigen #74104 Valencia, CA, USA) and the amount of RNA was measured with a Nanodrop spectrophotometer. Different samples were normalized to 1 μg total RNA, and DNA contamination was removed using Dnase1 AM treatment (Sigma #048K6043 St. Louis, MO, USA). After Dnase1 deactivation, reverse transcription was carried using a ISCUP™ cDNA synthesis kit (BIO-RAD #170-889 Hercules, CA, UAS1). Isoform specific primers with a T7 promoter in the 3'-end of the reverse primer were designed: TATTCCAGAAGACG and TAATACGACTCACTATAGGGAGAGCAAT for AT02763; CAAAGTCCTTCGTCAAACGC and TAATACGACTCACTATAGGGAGATTTCA for GH15753; TCAATTGCCAGGCAGATGGC and TAATACGACTCACTATAGGGAGTCACC for HL01565; GCCAACTCGGAGAAGTCG and TAATACGACTCACTATAGGGAGATTATG for LD28224; and GTGCAATTACCTGCCGTTTCG and TAATACGACTCACTATAGGGAGAGGAGA for GH08133.

The PCR reaction was then cleaned by phenol:chloroform extraction, and the DNA was ethanol precipitated and used as a template for *in situ *probes and hybridization of *Drosophila *embryos as described by Tear *et al*. [33]. The different probes were tagged with either fluoro-isothiocyanate (FITC), biotin, or Dig.

Mab 1D4, 24B10, anti-Eng, anti-Slit, anti-Eve, and BP102 antibodies were obtained from the Developmental Studies Hybridoma Bank and were used in a 1:5 dilution, while anti-mouse β-gal was purchased from Promega and used in a 1:10,000 dilution. Iowa city, Iowa, USA

*turtle *mutant chromosomes were balanced over a CyO, Elav-lacZ balancer. Homozygous mutant embryos were identified by the absence of anti-mouse β-gal staining. To quantify nerve defects in a given mutant genotype, eight to ten mutant embryos were fixed, dissected, and stained with anti-FasII. The detection of anti-FasII was achieved using an anti-mouse horseradish peroxidase-conjugated secondary antibody, detected by DAB/1:10 dilution 30% H_2_O_2 _staining. Only nerves from abdominal hemisegments A2 to A7 were scored.

For immunocytochemistry of adult heads and retinal axons, 5-day-old fly heads were dissected (proboscis and air sacs removed) and fixed for 2 hours in ice with 4% paraformaldehyde in NaCl-free 5× phosphate-buffered saline. They were then washed extensively with phosphate-buffered saline and cryosectioned. The sections were fixed again for 20 minutes in 4% paraformaldehyde, followed by another round of washing. Immunostaining with or without DAPI was then performed.

### Blind test

Wild-type and *tutl*^*01085 *^mutant embryos were collected, fixed simultaneously, and processed under the same conditions. They were dissected and stained with FasII. The embryos were mounted on slides, with each slide containing only one embryo. Samples were given random identification numbers. An independent observer was then asked to examine the slides and attempt to determine each embryo's genotype; the observer's evaluations were compared with the true genotypes of the embryos.

### Microscopy

For fluorescence detection, the specimen was imaged under a 20 × 1.4 numerical aperture lens on a Zeiss LSM 510 META confocal microscope. The DAPI channel (Excitation: λ 720 nm; Emission Band Pass 365 to 480 nm) and FITC channel (Excitation: λ 488 nm; Emission Band Pass 500 to 550 nm) were acquired. The acquired Z-stack was flattened as a maximum intensity projection and exported to a TIF file. Cropping and adjustment of brightness and contrast were performed in Adobe Photoshop.

### Transfection and maintenance of Schneider 2 cells

Schneider 2 (S2) cells were maintained at room temperature in a growth medium composed of Schneider medium with 10% fetal calf serum and penicillin/streptomycin (all reagents were from Gibco-Invitrogen Carlsbad, CA, USA). The cells were transfected using the Cell Infecting Kit (Invitrogen Carlsbad, CA, USA), as described by the manufacturer, and were maintained at room temperature in a growth medium containing 400 μg/ml Zeocin.

### SDS-PAGE and western blot analysis

Both conditioned medium and S2 cells were collected 1 week post-transfection. Conditioned medium (15 μl) and the S2 cell pellet were solubilized using SDS-polyacrylamide sample buffer. The samples were then run on 10% SDS-PAGE and transferred onto a nitrocellulose filter using standard procedures.

### EST clones and transgenes

cDNA clones of *turtle *were obtained from Research Genetics-Invitrogen Carlsbad, CA, USA. To subclone the His-tagged GH01573 and AT02763 transcripts into the PIZT insect expression vector (Invitrogen), we performed a standard PCR reaction using high-fidelity Pfu taq. Plasmid DNA of GH15753 and AT02763 (100 ng) was used as a template. Two primers were designed for each EST clone; the 5'-primers contained an *Eco*RI site before the initiation codon, while the 3'-end primers contained an in-frame His-tag sequence followed by a stop codon and an *Xba*I restriction site. The resulting PCR product was digested with *Eco*RI/*Xba*I overnight, and then ligated to a linearized *Eco*RI/*Xba*I digested PIZT vector. The ligation product was transfected into DH10B electrocompetent *Escherichia coli*, and successful transformants were selected based upon the Zeocin resistance conferred by the PIZT vector. Plasmids were isolated and sequenced by the Keck facility. Yale University, New Haven, CT, USA Only transformants that had the fully correct sequence of GH01573-His were used for S2 *Drosophila *cell line transfection.

Codon sequences GH15753, AT02763, HL010565, and LD28824 were subcloned into a PUAST vector and were used to transform *w*^1118 ^flies using standard transgenic procedures.

## Abbreviations

bp: base pair; *comm*: *commissureless*; DAPI: 4',6-diamidino-2-phenylindole; EGUF: eyeless-GAL4 UAS-FLP; EST: expressed sequence tag; Fas: Fasciclin; FITC: fluoro-isothiocyanate; *robo*: *roundabout*; S2: Schneider 2.

## Competing interests

Both Bader F Al-Anzi and Robert J Wyman declare that they have no competing interests that are defined as a set of conditions in which professional judgment concerning a primary interest (validity of research) is unduly influenced by a secondary interest (such as financial gain).

## Authors' contributions

First author and author of correspondence: Bader F Al-Anzi. Second author: Robert J Wyman.

## Supplementary Material

Additional file 1 **Extent of the genomic deficiency in *tutl*^*ex383*^**. **(A) **Southern blot of *Cla*I DNA digest using both 5' and 3' probes showing polymorphism with only the 3' probe (1 is wild type, 2 is *tutl*^*l*(2)*k14703*^, and 3 is *tutl*^*ex383*^). The location of PCR primers A and B series along the *tutl *gene (B), PCR reaction from DNA template isolated from *tutl*^*ex383 *^using A4/B2 primers produces a 900-bp DNA product (C). **(D) **The sequencing of this PCR product indicates the presence of an 11,430-bp deficiency in the *tutl*^*ex383 *^mutant allele. Click here for file

Additional file 2 **Lack of cell fate change in *turtle *mutants**. Panels are paired, wild-type at left and mutant at right. **(A, B) **Neuronal and midline cells stained with anti-Even-skipped. **(C, D) **Neuronal and midline cells stained with anti-Engrailed. **(E, F) **Late embryonic denticle bands. **(G, H) **Midline glia stained with anti-Slit. **(I-K) **Wild-type (I), *tutl*^*k14703 *^(J), and *turtle*-overexpressing (K) eye imaginal disc photoreceptors stained with 24B10. **(L, M) **Glia stained with anti-Repo. Click here for file

Additional file 3 **Quantification of the motor nerve defects seen in 55 to 60 A5 to A10 embryonic hemisegments after overexpressing the different *turtle *isoforms on a wild-type background**. **(A) **Overexpression using the pan-neuronal driver Sca-Gal4. **(B) **Overexpression using the skeletal muscle driver 24B-Gal4. Click here for file
