# Supplementary figures and images for: The Drosophila immunoglobulin gene turtle encodes guidance molecules involved in axon pathfinding
Source: Neural Dev. 2009 Aug 17;4:31. doi: 10.1186/1749-8104-4-31 (PMC2739522; doi:10.1186/1749-8104-4-31)

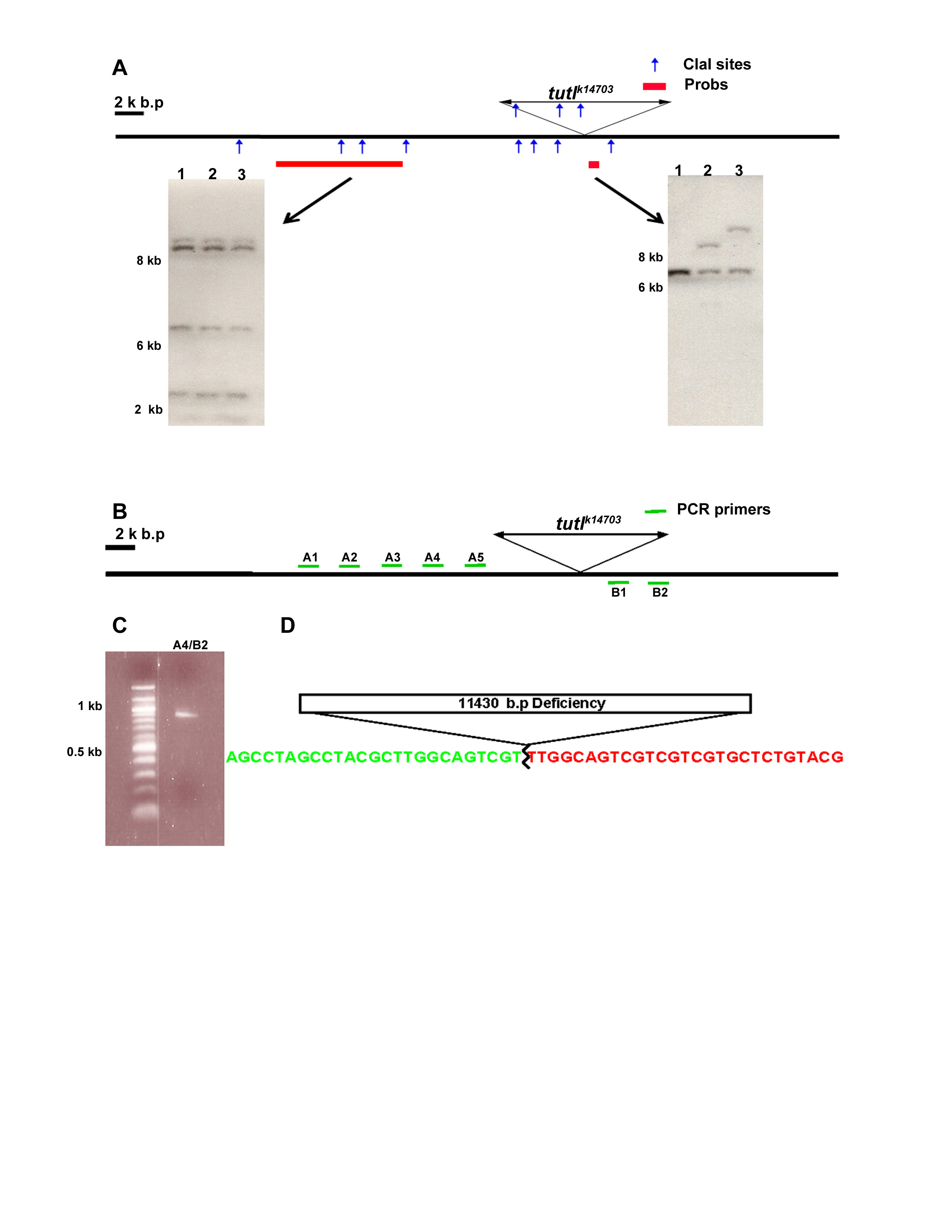

Supplement: Additional file 1 — Extent of the genomic deficiency in tutlex383. (A) Southern blot of ClaI DNA digest using both 5' and 3' probes showing polymorphism with only the 3' probe (1 is wild type, 2 is tutll(2)k14703, and 3 is tutlex383). The location of PCR primers A and B series along the tutl gene (B), PCR reaction from DNA template isolated from tutlex383 using A4/B2 primers produces a 900-bp DNA product (C). (D) The sequencing of this PCR product indicates the presence of an 11,430-bp deficiency in the tutlex383 mutant allele. [file 1749-8104-4-31-S1.jpeg]

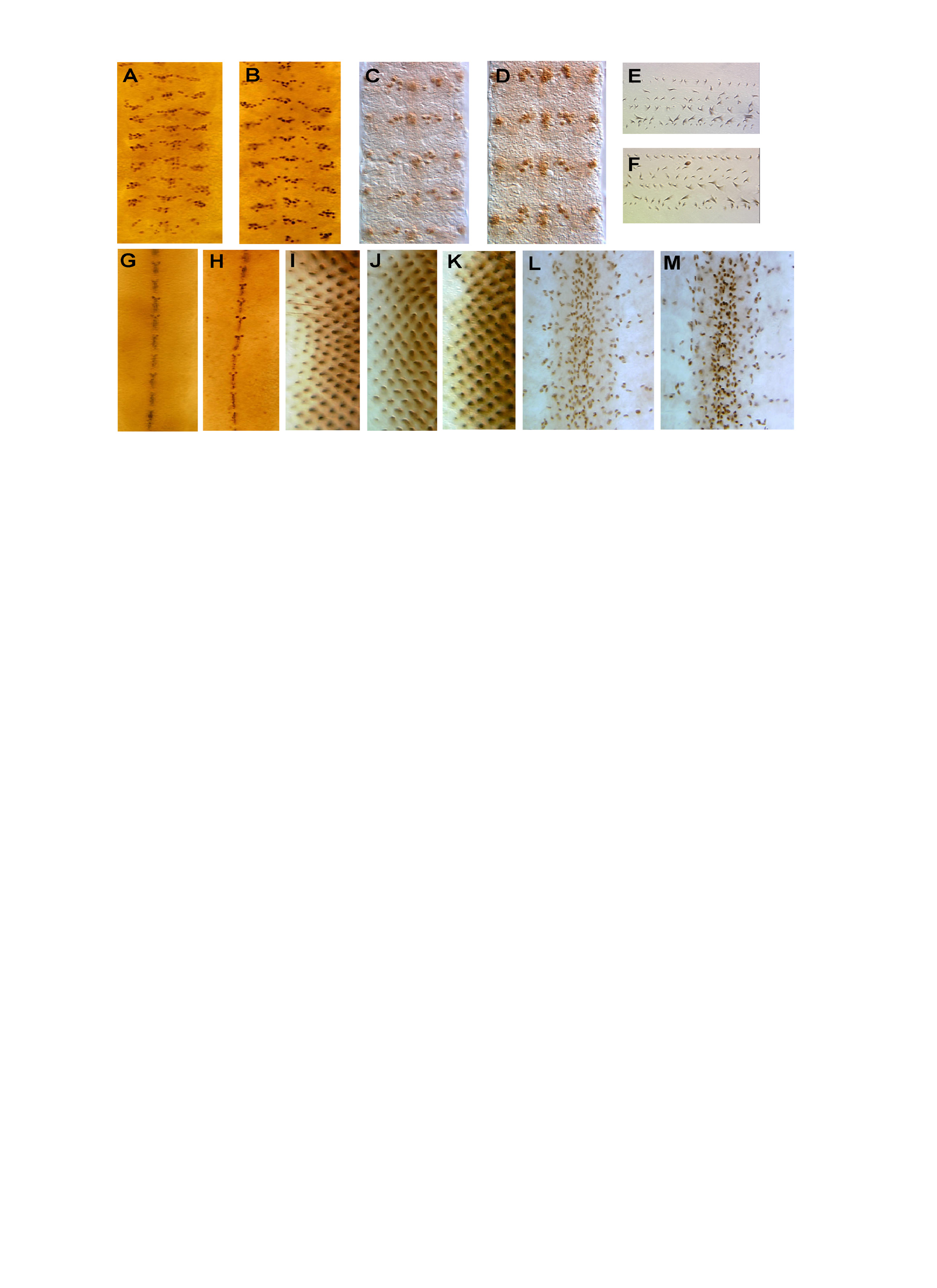

Supplement: Additional file 2 — Lack of cell fate change in turtle mutants. Panels are paired, wild-type at left and mutant at right. (A, B) Neuronal and midline cells stained with anti-Even-skipped. (C, D) Neuronal and midline cells stained with anti-Engrailed. (E, F) Late embryonic denticle bands. (G, H) Midline glia stained with anti-Slit. (I-K) Wild-type (I), tutlk14703 (J), and turtle-overexpressing (K) eye imaginal disc photoreceptors stained with 24B10. (L, M) Glia stained with anti-Repo. [file 1749-8104-4-31-S2.jpeg]

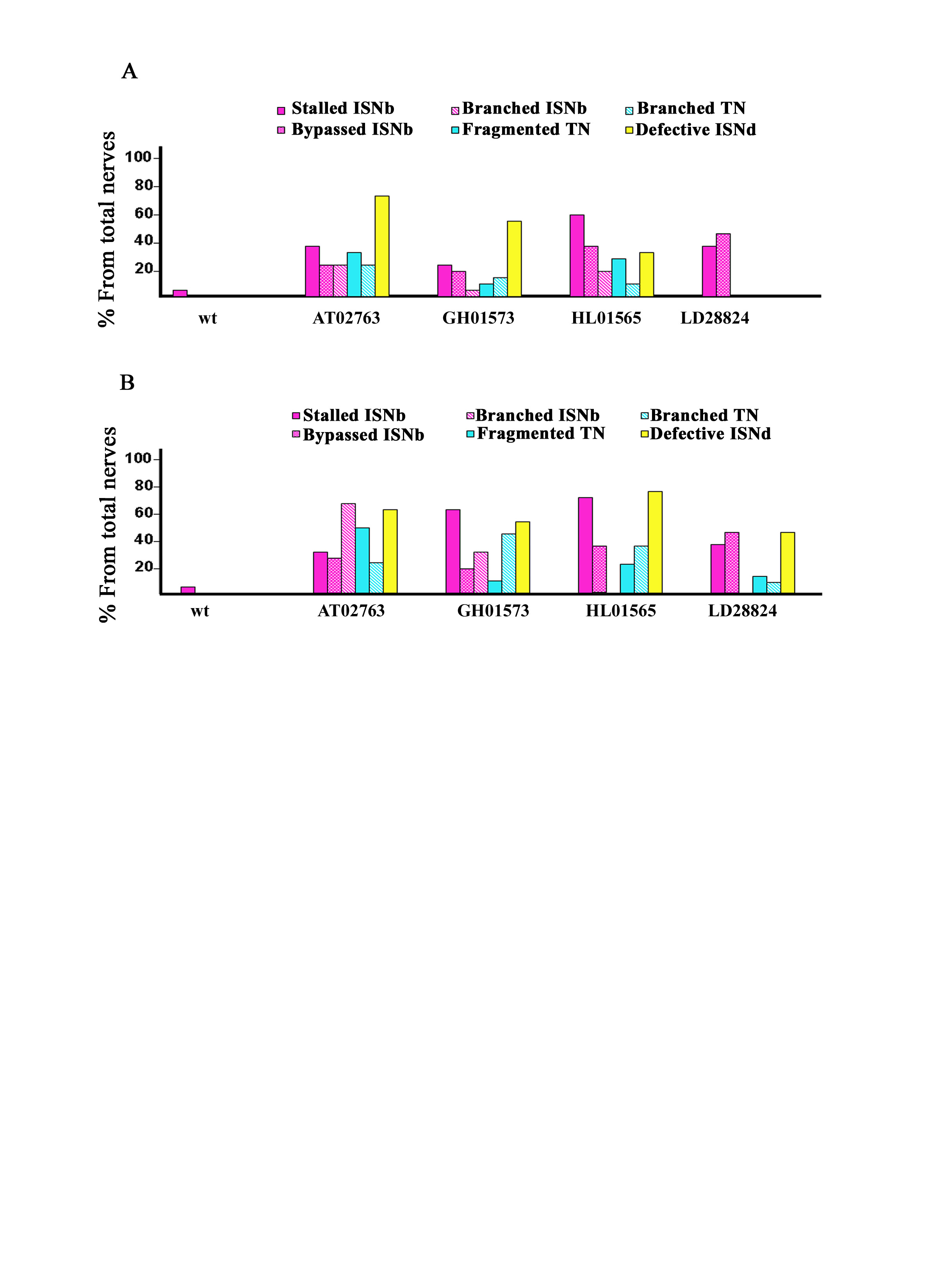

Supplement: Additional file 3 — Quantification of the motor nerve defects seen in 55 to 60 A5 to A10 embryonic hemisegments after overexpressing the different turtle isoforms on a wild-type background. (A) Overexpression using the pan-neuronal driver Sca-Gal4. (B) Overexpression using the skeletal muscle driver 24B-Gal4. [file 1749-8104-4-31-S3.jpeg]
